# Honey and Its Phenolic Compounds as an Effective Natural Medicine for Cardiovascular Diseases in Humans?

**DOI:** 10.3390/nu12020283

**Published:** 2020-01-21

**Authors:** Beata Olas

**Affiliations:** Faculty of Biology and Environmental Protection, Department of General Biochemistry, University of Lodz, Pomorska 141/3, 90-236 Lodz, Poland; beata.olas@biol.uni.lodz.pl; Tel./Fax: +48-4-2635-4484

**Keywords:** honey, blood platelet, antioxidant, coagulation, cardiovascular system

## Abstract

Honey is a sweet, viscous syrup produced by the honey bee (*Apis mellifera*). It is probably the first natural sweetener ever discovered, and is currently used as a nutritious food supplement and medicinal agent. The aim of the present mini-review is to summarize and update the current knowledge regarding the role of honey in CVDs based on various experimental models. It also describes the role of its phenolic compounds in treating CVDs. Many such phenolic and flavonoid compounds, including quercetin, kaempferol, apigenin, and caffeic acid, have antioxidant and anti-platelet potential, and hence may ameliorate cardiovascular diseases (CVDs) through various mechanisms, such as by decreasing oxidative stress and inhibiting blood platelet activation. However, as the phenolic content of a particular type of honey is not always known, it can be difficult to determine whether any observed effects on the human cardiovascular system may be associated with the consumption of honey or its constituents. Therefore, further experiments in this area are needed.

## 1. Introduction

Of all the lifestyle factors that play an important role in human health, nutrition is key. Recent clinical and epidemiological studies indicate that honey may act as an important mediator of human health [1,2,3,4,5,6,7,8,9].

Honey, a natural substance which has been used as a sweetener for thousands of years, is produced from honeydew or flower nectar by bees (*Apis mellifere*). Its chemical composition is influenced by its botanical origin, mode of processing, seasons, and environmental conditions [7,10]. Honey is not only considered a food or a sweetener, its consumption has long been regarded as having beneficial effects on human health, as described in early Greek, Roman, Vedic, and Islamic texts [11]. In addition, more recent in vitro and in vivo studies have confirmed that honey possesses a range of antioxidant, antimicrobial, antiviral, anticancer, and antidiabetic properties, and it has been shown to demonstrate protective activities on the nervous, cardiovascular, gastrointestinal, and respiratory systems [1,2,3,6,7].

Most of the biological activities of honey are attributed to its constituent phenolic and flavonoid compounds. It has been found that the effect of honey on the cardiovascular system depends on the bioavailability of various phytochemical compounds, and on their methods of absorption and metabolization [7]. This is not unexpected, as each type of honey bestows individual health benefits [12,13,14].

Frankel et al. [15] report that the color of honey is related to its content of various plant pigments, especially carotenoids. Khalil and Sulaiman [1] also describe a correlation between color and antioxidant capacity, with darker honeys having the highest levels of antioxidants. Gheldof et al. [16] also note a significant correlation between the phenolic concentration of honey and its antioxidant activity measured in vitro as lipoprotein oxidation of human plasma. Similar correlations have been observed by other authors [17,18].

Honey is produced almost all over the world, with global production estimated to be approximately 1.2 million tons [19]. Consumption varies worldwide, from 0.3–0.4 kg per capita in Italy or France, to 1.0 to 1.8 kg per capita in Germany or Austria [19]. As current recommendations for the use of honey in the prophylaxis and treatment of cardiovascular diseases (CVDs) in humans are based on case reports and small clinical experiments, the aim of the present mini-review is to summarize the present state of knowledge regarding the role of honey in treating CVDs, as derived from in vitro and in vivo studies based on both animals and humans. It also describes the effects of its constituent phenolic and flavonoid compounds on CVDs. Its findings may have important implications for the prophylaxis and treatment of CVDs, especially in humans.

## 2. Chemical Compounds Present in Honey

Honey contains about 180 different compounds, including organic acids, trace elements, minerals, vitamins, enzymes, and proteins; however, its primary group of compounds by weight are carbohydrates, comprising approximately 26 mono- and di-saccharides, typically present as 64.9 to 73.1 g/100 g of honey, and fructose, constituting 35.6 to 41.8 g/100 g of honey. These sugar components determine the energy value: 100 g of honey provides about 300 kcal, with a daily dose of 20 g covering about 3% of the recommended daily calorie intake [20,21,22].

Honey is also a natural source of flavonoids, phenolic acids, and derivatives of phenolic acids [1,17,18,23,24,25,26,27,28,29,30,31,32,33,34,35], and the phenolic content of honey is believed to determine its biological properties, especially its antioxidant potential. Furthermore, acaetin, caffeic acid, quercetin, galangin, and kaempferol, as well as other phenolic compounds present in honey, may offer promise as pharmaceuticals in the treatment of cardiovascular diseases [1]. In fact, according to the United States Department of Agriculture (USDA) database, mixed verities of honey samples from various countries contain apigenin (0.03 mg/100 g), isorhamnetin (0.06 mg/100 g), kaempferol (0.06 mg/100 g), luteolin (0.28 mg/100 g), quercetin (0.31 mg/100 g), and myricetin (0.36 mg/100 g) [11]. Of the various types of techniques available to identify honey phenolic and flavonoid compounds, the most commonly used one is high-performance liquid chromatography (HPLC) [36].

Honey also includes several antioxidant enzymes, such as catalase and glucose oxidase, in its composition [37]. The most commonly-found phenolic compounds in selected honeys are given in Table 1. Interestingly, acacia honey, manuka honey, and eucalyptus honey are better sources of flavonoids than sunflower honey, while lavender honey and orange honey are sole sources of certain phenolic acids (Table 1). Phenolic compounds typically constitute approximately 56–500 mg per kg of honey, with the concentration ranging from 60 to 460 mg/100 g of honey [38,39].

A study of the phenolic acid profiles of 12 honeys collected from various regions in Greece found them to be rich in phenolic acids, in particular, protocatechuic acid and p-hydroxybenzoic acid [40]. In addition, interesting variations in honey content were observed with regard to the source of honey. For example, significantly higher concentrations of protocatechuic acid were found in pine and fir honey (mean—6640 and 397 µg/kg honey, respectively) than thyme and citrus honey (mean—437.6 and 116 µg/kg, respectively), while p-Hydroxybenzoic acid (mean—1252.5 µg/kg honey) was the dominant phenolic compound in thyme honeys [40]. Some studies have proposed chemical markers for determining the botanical origin of honey based on the presence and abundance of one or more specific phenolic compounds [41]. Ferreres et al. [42] suggest that ellagic acid may serve as a suitable marker for *Erica* spp. (heather) from Portugal.

Few papers have been published on the bioavailability and metabolism of the phenolic and flavonoid compounds in honey. Schramm et al. [43] report that plasma total phenolic concentration increased together with the antioxidant and reducing capacities of plasma after consumption of 15 g of honey/kg bodyweight for two types of honey. Forty subjects took part in this experiment. However, the authors did not describe how long the subjects consumed honey in this study. Recently, it has also been reported that the absorption and metabolism of honey phenolic compounds takes place in the small intestine [2].

Bogdanow et al. [19] indicate that honey can contain nitric oxide metabolites, which may play a protective role against CVDs. The chemical composition of honey, as well as the bioavailability and metabolism of its constituents, has been described in a number of review papers [1,2,3,5,6,7,8,9].

## 3. Honey and Cardiovascular Diseases

Cardiovascular diseases (CVDs) constitute a serious challenge for modern medicine. Studies indicate that certain factors, such as oxidative stress, hypertension, hypercholesterolemia, inflammatory factors, and diabetes play particularly significant roles in their development. In addition, obesity, physical inactivity, and the increased blood clotting and hyperactivation of blood platelets may also play important roles [44,45,46]. However, clinical trials with antioxidant supplementation do not always demonstrate efficacy for CVD reduction. On the other hand, there are few papers which describe the role of antioxidants in CVD reduction. For example, Daskalova et al. [47] reported that commercial aronia berry products are a rich source of phenolic compounds, and it has antioxidant properties. In addition, it has anti-atherogenic and cardioprotective effects in aging rats.

### 3.1. In Vitro Experiments

Ahmed et al. [48] examined the effect of honey in vitro on various elements of hemostasis: blood platelet functions (measured by platelet aggregation) and coagulation processes, measured as activated partial prothrombin time (APTT), prothrombin time (PT), thrombin time (TT), and fibrinogen level. They found honey to inhibit platelet aggregation with an IC_50_ of 5 to 7.5%, and to prolong APPT, PT, and TT at concentrations of 3.75% and 7.5%. Incubation with 6.25% and 11.75% honey also reduced the fibrinogen concentration of platelet-poor plasma: a 13% reduction was observed following the addition of 6.25% honey compared to control samples without honey.

Interestingly, Rossiter et al. [49] report that certain medical honey preparations, viz. medicinal honey (Activon), supermarket honey (Rowse), and honey-based ointment (Mesitran), promote angiogenetic properties in rat aortic ring assay in vitro. In addition, medicinal honey demonstrated greater activity than supermarket honey (Rowse).

Kim et al. [50] suggest that honey demonstrates anti-platelet and antithrombotic effects when used as a component of Kyuang-OK-KO (KOK), measured as inhibition of collagen-induced platelet aggregation and shape change. They propose that the anti-platelet action of KOK acts through the inhibition of ATP release, intracellular Ca^2+^ elevation, and phosphorylation of PLCγ and protein kinase B (Akt). KOK not only demonstrated anti-platelet properties in an in vitro model based on rat blood platelets, but also prevented thrombosis in mice. The in vitro model used KOK at concentrations of 0.3, 1.3, and 10 mg/mL, while the in vivo model used it at 2 g/kg for one day, or 0.5, 1, or 2 g/kg for seven days. However, it is very difficult to demonstrate the anti-platelet and antithrombotic actions of honey, because authors used a food mixture that contains honey as well as other ingredients.

In the aforementioned study of 12 types of Greek honey, Spilioti et al. [40] evaluated antioxidant potential using the oxygen radical absorbance capacity (ORAC) test. ORAC values ranged from 619–2129 µmol Trolox equivalent (TE)/kg for pine and fir tree honeys, and from 415–692 µmol TE/kg for citrus and thyme honeys. The honeys were also found to have antiatherogenic properties: for example, when administered at 20–500 µg/mL, honey reduced the expression of adhesion molecules vascular cell adhesive protein 1 (VCAM-1) and intercellular adhesion molecule 1 (ICAM-1) in endothelial cells. This activity was also found to be associated with phenolic acid content.

### 3.2. In Vivo Experiments

In vitro experiments have shown that honey has cardioprotective potential; however, there are only preliminary results. In vivo experiments partly have also demonstrated this potential. James et al. [51] describe the role of herbal medicine in hypertensive patients (260 study participants). The most commonly used herbal medicine among users were honey (n = 89; 33.3%), moringa (n = 80, 30.0%, and garlic (n = 73, 27.3%). However, they report that honey consumption has a beneficial effect (especially diabetes mellitus and weight reduction) in hypertensive patients. Moreover, the authors do not describe the type or chemical characteristics of the tested honey.

Rakha et al. [52] found that pretreatment with natural wild honey (5 g/kg) for one hour prior to injection with epinephrine (100 µg/kg) protects anesthetized normal rats from the incidence of epinephrine-induced cardiac disorders and vasomotor dysfunction.

Khalil et al. [53] found Tualang honey to exert a cardioprotective effect by inhibiting oxidative stress, as determined by the content of thiobarbituric acid reactive substances (TBARS), a marker of lipid peroxidation, and the activity of antioxidant enzymes. In this experiment, 40 male albino Wistar rats received Tualang honey (3 g/kg/day) orally for 45 days.

Afroz et al. [54] report that Sundarban honey (5 mg/kg/day, for six weeks) confers protection against isoproterenol-induced myocardial infarction in Wistar rats. The mechanism of action of the honey was associated with inhibition of oxidative stress, measured by the TBARS content in rat heart tissues. Sundarban honey, one of the most renowned types of honey from Bangladesh, is a wild multi-floral honey produced by *Apis dorsata* bees. In addition, Islam et al. [55] note that Sundarban honey contains higher levels of phenolic compounds than other Bangladeshi honeys, with a content of about 690 mg gallic acid/kg.

Erejuwa et al. [56] found that Nigerian honey (1 and 2 g/kg) ameliorated hyperlipidemia in a group of alloxan-induced diabetic rats. Honey was administered to diabetic rats for three weeks. After this time, high-density lipoprotein cholesterol (HDL cholesterol) was found to be increased, while triglyceride and very low density lipoprotein cholesterol (VLDL cholesterol) levels were decreased. Moreover, honey (1 and 2 g/kg) significantly reduced hyperglycemia. Compared with the initial blood glucose level was lower (about 40%) in diabetic rats administered 1 g/kg body weight of honey.

Rasad et al. [57] compared the effect of honey consumption and sucrose consumption on lipid profile in young healthy people. One group received 70 g per day of natural honey mixed with 250 mL of tap water. A second group received 70 g per day of sucrose mixed with 250 mL of tap water. The lipid profile was determined after six weeks. It was found that honey consumption improved the lipid profile, consisting of total cholesterol, triglycerides (TG), and low-density lipoprotein cholesterol (LDL cholesterol), and increased HDL cholesterol. The reverse was observed for consumption of sucrose. The authors suggest that the effects of the honey may be associated with the presence of trace elements which act as antioxidants; in addition, the honey also increased lipid synthesis and reduced lipolysis, which can lower the level of lipids in the serum. Other authors observed that 2 weeks of daily consumption of 50 g carbohydrate from sucrose and honey exerted similar effects on measures of glycemia, inflammation, and lipid status in glucose-tolerant and intolerant individuals [58].

Both in vitro and animal in vivo experiments have demonstrated that honey can serve as a natural treatment for CVDs through its antioxidant and antiplatelet properties, as well as its other biological potential (Table 2); however, only one experiment has been performed in vivo in a human model, and this was based on a group of young, healthy people. In addition, the study does not define the type of tested honey or its chemical content [57].

## 4. Honey Phenolics and Cardiovascular Diseases

Phenolic compounds are ubiquitous throughout the plant kingdom and are readily incorporated into honey via nectar or pollen acquired from plants visited by the honeybee [2]. The protective effects of the phenolic compounds present in honey may be beneficial for the prophylaxis and treatment of cardiovascular diseases. Such beneficial effects include antioxidant, anti-platelet, and vasorelaxant properties [60,61]. Recent evidence indicates the presence of about thirty types of phenolic compounds in honey; however, the profile of these phenolic compounds can depend on various factors, including the floral source, and the climatic and geographical conditions. For example, quercetin, galangin, kaempferol, isorhamnetin, and luteolin are present in all types of honey, but hesperetin and naringenin are found only in specific varieties [62,63]. Studies have found flavonoids to exert beneficial actions on the cardiovascular system via inhibition of blood platelet activation, reduction of LDL cholesterol level, and by exerting anti-inflammatory activity. Tuberoso et al. [64] report that bitter strawberry-tree honey obtained from strawberry-tree flowers (*Arbutus unedo* L.) did not induce vasodilation, even at the highest tested concentration (0.206 g/L), despite it containing high concentrations of phenolic compounds: about 920 mg gallic acid equivalent (GAE)/kg. Guerrero et al. [65] note that honey phenolic compounds such as apigenin, quercetin, catechin, and luteolin inhibit blood platelet aggregation though binding to the thromboxane A_2_ receptor in an in vitro model.

Chrysin is a natural flavonoid present in high concentrations in various types of honey. It is also present in propolis, various fruits, vegetables, and plant extracts. Anghel et al. [66] report that chrysin (50 mg/kg, daily) inhibits methotrexate (MTX)—triggered cardiomyocyte apoptosis via various pathways, for example by decrease of Bax/Bcl-2 ratio and reducing caspase-3 expression. Farkhondeh et al. [67] also found chrysin to have a number of cardioprotective effects. It acted as an antioxidant, decreased lipid synthesis, and increased lipid metabolism. It also modulated vascular function by increasing the bioavailability of nitric oxide, inhibited the development of atherosclerosis by reducing vascular inflammation, and prevented vascular smooth muscle cell proliferation and thrombogenesis. Its anti-inflammatory action correlated with inhibition of the nuclear factor-κB signaling pathway.

Gang et al. [68] also found chrysin to demonstrate anti-platelet activity by inhibiting the blood platelet αIIbβ3-mediated signaling pathway in vitro. They report that five minute incubation with 1–100 µM chrysin reduced blood platelet aggregation, measured using an aggregometer, and granule secretion, measured by ATP release using luciferin/luciferase reagent, in a dose-dependent manner. Both processes were stimulated by various agonists, including 1 µg/mL collagen. It also inhibited platelet adhesion to fibrinogen, as indicated by fluorescence microscopy, and suppressed the phosphoinositide 3-kinase (PI3K)/Akt/glycogen synthase kinase 3β (GSK3β) signaling and spleen tyrosine kinase (Syk)—phospholipase Cγ (PLCγ)—protein kinase C (PKC) signaling cascades in blood platelets, both of which play a role in clotting.

Ravishankar et al. [69] found ruthenium-conjugated chrysin to potentially serve as a promising template for the development of novel anti-thrombotic agents. This conjugate inhibited blood platelet functions and thrombus formation in vitro, and did not exert cytotoxic effects on blood platelets, as determined by the lactate dehydrogenase assay. The tested conjugate was used at concentrations between 6.25 and 100 µM.

The most widely distributed flavonoid in foods, including honey, is quercetin. Most of the quercetin present in plants is attached to sugar moieties rather than in the free form [70,71]. Various studies indicate that quercetin lowers blood pressure and restores endothelial dysfunction in hypertensive animals. Quercetin has been found to downregulate NADPH oxidase, increase endothelial nitric oxide synthase (eNOS) activity, and prevent endothelial dysfunction in spontaneously hypertensive male rats when applied for 13 weeks at 10 mg/kg bodyweight [72]. On the other hand, Carlstrom et al. [73] suggest that a diet high in quercetin is not associated with a reduced risk of developing cardiovascular diseases. Their study examined vascular dysfunction, hypertension, and cardiac hypertrophy in hypertensive rats given a diet supplemented with quercetin (1.5 g quercetin/kg diet) for five or 11 weeks.

Duarte et al. [74] note a decreased risk of developing CVDs in hypertensive rats given a diet enriched with quercetin (10 mg/kg daily) for five weeks. This reduction was correlated with lowered oxidative stress. The level of oxidative stress was measured according to urinary isoprostane F_2_ and plasma malonyldialdehyde (MDA) levels. In addition, quercetin supplementation has been found to reduce blood pressure in hypertensive humans and to reduce oxidative stress by scavenging reactive oxygen species (ROS), inhibiting xanthine oxidase, chelating metal ions, and reducing lipid peroxidation in vitro [75].

The mitogen-activated protein kinase (MAPK) pathway is known to be involved in various cardiovascular diseases. Min et al. [76] indicate that quercetin inhibits ROS activation of the MAPK pathway. A similar effect was observed by Wu et al. [77].

Recently, Nie et al. [78] have observed that quercetin reduces atherosclerotic lesions by altering the gut microbiota and reducing atherogenic lipid metabolites. In this experiment, mice were maintained on a high-fat diet with or without oral quercetin supplementation for 12 weeks. The findings indicate that quercetin reduces oxidative stress, measured by MDA, and inhibits inflammatory processes, measured by the level of interleukin 6. In addition, quercetin decreased the levels of cholesterol and lysophosphatidic acids, and altered the composition of the gut microbiota.

As 500 mg of quercetin, in the aglycone form, is believed to be the optimal dose for lowering blood pressure and reducing inflammation processes, it is entirely possible that quercetin-rich plants and various food products have cardiovascular potential [70,71].

Kaempferol, like quercetin, has been found to possess protective effects against CVD via three general mechanisms: suppression of TNF-α production and activation of NF-κB, activation of Ca^2+^-activated K^+^ channels and increasing endothelial NOS activity by stimulating arterial relaxation, and reduction of oxidative stress. Kaempherol has also been found to inhibit the expression of cyclooxygenase [5,79].

Another common flavonoid in honey that may have an important role in the treatment of CVDs is luteolin. Li et al. [80] report that luteolin protects against diabetic cardiomyopathy by reducing the expression of matrix proteins and cellular hypertrophy. It does so by inhibiting NF-κB-mediated inflammation and activating the nuclear factor-erythroid 2 related factor 2 (Nrf2) antioxidant response. It also prevented cardiac fibrosis and hypertrophy. Oyagbemi et al. [81] indicate that the protective mechanism action of luteolin is also associated with the NF-κB/Nrf2 signaling pathways. They note that daily supplementation with 100 mg/kg and 200 mg/kg luteolin reduced high blood pressure and oxidative stress, including lipid peroxidation (expressed as MDA) and protein carbonylation, among 40 male Wistar albino rats. Another study by Ou et al. [82] found luteolin to have antioxidant potential against various parameters of oxidative stress, to protect against oxidative stress induced by hydrogen peroxide, a hydrogen radical donor, and reduce the production of ROS in human umbilical vein endothelial cells (HUVECs). The authors propose that luteolin may exert its effects through the regulation of protein kinase C. Luteolin has also been found to display inhibitory effects on the proliferation and migration of vascular smooth muscle cells, suggesting that it may also be a potential candidate for preventing and treating atherosclerosis [83].

Another flavonoid found in honey is pinocembrin, and this too has been observed to have various antioxidant, antimicrobial, and anti-inflammatory properties. Lungkaphin et al. [84] found it to reduce cardiac arrythmia and infarct size in rats subjected to acute myocardial ischemia/reperfusion. In this study, 20 male Wistar rats were randomly divided into two groups to receive either pinocembrin (30 mg/kg body weight) or the vehicle intravenously. Thirty minutes later, the left anterior descending coronary artery of each rat was ligated for 30 min, and then reperfusion was allowed for 120 min. The pinocembrin group demonstrated decreased MDA concentration and Bax/Bcl-2 ratio, and an elevated phosphorylated connexin 43 to total connexin ratio in the infarcted area. Sang et al. [85] also report that the combination of pinocembrin (20 mg/kg per day) and simvastatin (10 mg/kg per day) synergistically reduced atherosclerotic lesion development in eight-week-old male ApoE-/-mice with hyperlipidemia, possibly due in part to its protective effect on the vascular endothelium.

Recently, Wang et al. [86] have suggested that galangin may also protect against cardiac remodeling by decreasing inflammatory responses and apoptosis. Its action was associated with the inhibition of PI3K-Akt-GSK3β signaling pathways.

Other observations have shown that the phenolic acids in honey may play an important role in the prophylaxis and treatment of CVDs. For example, caffeic acid reduces lipid peroxidation and increases vitamin E levels in plasma in iron-overloaded rats [87].

It is important to note that it is unknown whether pure phenolic compounds isolated from honey are more effective for prophylaxis and treatment of CVDs than the honey itself. In addition, use doses of pure phenolic compounds are very often greater than in honey. Therefore, these types of studies do not make a compelling case for possible benefits of honey.

## 5. Adverse Effects of Honey

Despite its medicinal and nutritional value, honey is prone to microbial and non-microbial contamination. It also has traces of pesticides, herbicides, and heavy metals, obtained from the environment, and antibiotics administered by beekeepers [88]. In addition, honey may contain poisonous compounds, for example, the grayanotoxins found in mad honey from *Andromeda* flowers. Yaylaci et al. [89], Dur et al. [90], Karabag et al. [91], and Erenler [92] report that certain cardiac effects, such as bradycardia, asystole, acute myocardial infarction, and nodal rhythm, are associated with mad honey poisoning. Therefore, to ensure the safety of honey production, it has to comply with certain standard protocols and legislation.

## 6. Conclusions

Honeys exerts a protective effect against CVDs, and this activity is dependent on multiple factors, one of which is its chemical content, especially its constituent phenolic compounds. While the antioxidant properties of honey have been found to correlate with total phenolic compound content, they most likely arise as a result of the synergic effect of the various phenolic compounds present [16,93,94,95,96]. A similar relationship may exist for other biological properties of honey, such as the anti-platelet potential of its phenolic compounds, which play a significant role in the prophylaxis and treatment of stroke, atherosclerosis, coronary heart disease, and other CVDs induced by hyperactivation of blood platelets. However, this review, examining whether honey is an effective medicine for CVDs in humans, has some limitations. Firstly, while it includes a number of studies based on in vitro and animal models, only a few were performed on human subjects, and the cardioprotective effect of honey remains relatively unstudied among humans with high risk factors, including those with obesity and high blood pressure. In addition, only few papers describe the chemical characteristics of honey, especially its phenolic compound profile, and its effects on CVDs. Therefore, it is difficult to clearly identify the role of honey and its phenolic compounds in CVDs and whether these compounds have potential side effects such as bleeding, tissue ischemia, and hypertension in humans. In conclusion, although pure phenolic compounds are known to demonstrate antioxidant and often anti-platelet properties, it is necessary to first identify all the other potential mechanisms mediated by honey phenolics before the true value of honey can be recognized as a potential prophylactic and therapeutic agent for CVDs, especially in humans. It is an important that the source of honey has a large impact on potential outcomes. Therefore, especially the chemical content of honeys (for example concentration of phenolic compounds) should be studied.

## Figures and Tables

**Table 1:** The most common phenolic compounds (flavonoids and phenolic acids) in different types of honey [7].

Phenolic Compounds	
Flavonoids	Phenolic Acids
**Eucalyptus Honey**	
Chrysin (C_15_H_10_O_4_)	Benzoic acid (C_7_H_6_O_2_)
Isorhamnetin (C_16_H_12_O_7_)	Caffeic acid (C_9_H_8_O_4_)
Kaempferol (C_15_H_10_O_6_)	Chlorogenic acid (C_16_H_18_O_9_)
Luteolin (C_15_H_10_O_6_)	Ellagic acid (C_14_H_6_O_8_)
Myricetin (C_15_H_10_O_8_)	Ferulic acid (C_10_H_10_O_4_)
Pinobanksin (C_15_H_12_O_5_)	Gallic acid (C_7_H_6_O_5_)
Pinocembrin (C_15_H_12_O_4_)	p-coumaric acid (C_9_H_8_O_3_)
Tricetin (C_15_H_10_O_7_)	Protocatechuic acid (C_7_H_6_O_4_)
Quercetin (C_15_H_10_O_7_)	Syringic acid (C_9_H_10_O_5_)
	Vanillic acid (C_8_H_8_O_4_)
**Manuka Honey**	
Chrysin (C_15_H_10_O_4_)	Caffeic acid (C_9_H_8_O_4_)
Galangin (C_15_H_10_O_5_)	Ferulic acid (C_10_H_10_O_4_)
Isorhamnetin (C_16_H_12_O_7_)	Gallic acid (C_7_H_6_O_5_)
Kaempferol (C_15_H_10_O_6_)	Syringic acid (C_9_H_10_O_5_)
Luteolin (C_15_H_10_O_6_)	
Pinobanksin (C_15_H_12_O_5_)	
Pinocembrin (C_15_H_12_O_4_)	
Quercetin (C_15_H_10_O_7_)	
**Acacia Honey**	
Apigenin (C_15_H_10_O_5_)	Caffeic acid (C_9_H_8_O_4_)
Chrysin (C_15_H_10_O_4_)	Chlorogenic acid (C_16_H_18_O_9_)
Galangin (C_15_H_10_O_5_)	Ferulic acid (C_10_H_10_O_4_)
Genistein (C_15_H_10_O_5_)	Gallic acid (C_7_H_6_O_5_)
Kaempferol (C_15_H_10_O_6_)	Syringic acid (C_9_H_10_O_5_)
Luteolin (C_15_H_10_O_6_)	Vanillic acid (C_8_H_8_O_4_)
Myricetin (C_15_H_10_O_8_)	
Pinobanksin (C_15_H_12_O_5_)	
Pinocembrin (C_15_H_12_O_4_)	
Quercetin (C_15_H_10_O_7_)	
**Sunflower Honey**	
Galangin (C_15_H_10_O_5_)	Benzoic acid (C_7_H_6_O_2_)
Pinobanksin (C_15_H_12_O_5_)	Caffeic acid (C_9_H_8_O_4_)
Pinocembrin (C_15_H_12_O_4_)	Ferulic acid (C_10_H_10_O_4_)
	Gallic acid (C_7_H_6_O_5_)
	p-coumaric acid (C_9_H_8_O_3_)
	Protocatechuic acid (C_7_H_6_O_4_)
	Syringic acid (C_9_H_10_O_5_)
	Vanillic acid (C_8_H_8_O_4_)
**Lavender Honey**	
	Benzoic acid (C_7_H_6_O_2_)
	Caffeic acid (C_9_H_8_O_4_)
	Ferulic acid (C_10_H_10_O_4_)
	Gallic acid (C_7_H_6_O_5_)
	p-coumaric acid (C_9_H_8_O_3_)
	Protocatechuic acid (C_7_H_6_O_4_)
	Syringic acid (C_9_H_10_O_5_)
	Vanillic acid (C_8_H_8_O_4_)
**Orange Honey**	
	Benzoic acid (C_7_H_6_O_2_)
	Caffeic acid (C_9_H_8_O_4_)
	Ferulic acid (C_10_H_10_O_4_)
	Gallic acid (C_7_H_6_O_5_)
	p-coumaric acid (C_9_H_8_O_3_)
	Protocatechuic acid (C_7_H_6_O_4_)
	Vanillic acid (C_8_H_8_O_4_)
**Pine Honey**	
Catechin (C_15_H_15_O_6_)	Protocatechuic acid (C_7_H_6_O_4_)

**Table 2:** Cardioprotective potential of honey in various experimental models.

Honey	Investigated Roles	References
Honey (chemical content: undefined)	Antioxidant effect (in vitro)	[16]
Honey (chemical content: undefined)	Anti-platelet and anticoagulant effects (in vitro)	[48]
Medical honey preparations (chemical content: unidentified)	Angiogenetic effect (in vitro)	[49]
Honey (chemical content: undefined)	Anti-platelet effect (in vitro)	[50]
Greek honeys (for example, the total phenolic compounds—5.2 ± 0.2 g GA/kg honey, for fir honey from Karpeniri)	Antiatherogenic effect (in vitro)	[40]
Honey (chemical content: undefined)	Antithrombotic effect (mice model)	[50]
Tualang honey (chemical content: undefined)	Antioxidative potential (rat model)	[59]
Sundarban honey (chemical content: undefined)	Antioxidative potential (rat model)	[54]
Nigerian honey (chemical content: undefined)	Modifying lipid metabolism (diabetic rat model)	[56]
Honey (chemical content: undefined)	Modifying lipid metabolism (in young healthy people)	[57]

## Abbreviations

ADP	adenosine diphosphate
Akt	protein kinase B
APPT	activated partial prothrombin time
CVD	cardiovascular disease
eNOS	endothelial nitric oxide synthase
GAE	gallic acid equivalent
GSK3β	glycogen synthase kinase 3β
HDL	high density lipoprotein
ICAM-1	intercellular adhesion molecule 1
KOK	Kyuang-OK-KO
LDL	low density lipoprotein
MAPK	mitogen-activated protein kinase
MDA	malonyldialdehyde
MTX	methotrexate
Nrf2	erythroid 2 related factor 2
ORAC	oxygen radical absorbance capacity
PKC	protein kinase C
PLCγ	phospholipase Cγ
PT	prothrombin time
ROS	reactive oxygen species
TBARS	thiobarbituric acid reactive substances
TE	Trolox equivalent
TG	triglycerides
TNF-α	tumor necrosis factor α
TRAP	thrombin receptor-activated peptide
TT	thrombin time
VCAM-1	vascular cell adhesive protein 1
VLDL	very low-density lipoprotein.

## References

[1] Khalil M.I., Sulaiman S.A. (2010). The potential role of honey and its polyphenols in preventing heart diseases: A review. Afr. J. Tradit. Complement. Altern. Med..

[2] Alvarez-Suarez J.M., Giampieri F., Battino M. (2013). Honey as a source of dietary antioxidants: Structures, bioavailability and evidence of protective effects against human chronic diseases. Curr. Med. Chem..

[3] Eteraf-Oskouei T., Najafi M. (2013). Traditional and modern uses of natural honey in human diseases: A review. Iran. J. Basic Med. Sci..

[4] Zpou P. (2016). Traditional Chinese medicine, food therapy, and hypertension control: A narrative review of Chinese literature. Am. J. Chin. Med..

[5] Miguel M.G., Antunes M.D., Faleiro M.L. (2017). Honey as a Complementary Medicine. Integr. Med. Insights.

[6] Samarghandian S., Farkhondeh T., Samini F. (2017). Honey and health: A review of recent clinical research. Pharmacogn. Res..

[7] Cianciosi D., Forbes-Hernández T.Y., Afrin S., Gasparrini M., Reboredo-Rodriguez P., Manna P.P., Zhang J., Lamas L.B., Flórez S.M., Toyos P.A. (2018). Phenolic Compounds in Honey and Their Associated Health Benefits: A Review. Molecules.

[8] Ramli N.Z., Chin K.Y., Zarkasi K.A., Ahmad F. (2018). A review on the protective effects of honey against metabolic syndrome. Nutrients.

[9] Nguyen H.T.L., Panyoyai N., Kasapis S., Pang E., Mantri N. (2019). Honey and its role in relieving multiple facets of atherosclerosis. Nutrients.

[10] Khan R.U., Naz S., Abudabos A.M. (2017). Towards a better understanding of the therapeutic applications and corresponding mechanisms of action of honey. Environ. Sci. Pollut. Res..

[11] Hossen M.S., Ali M.Y., Jahurul M.H.A., Abdel-Daim M.M., Gan S.H., Khalil M.I. (2017). Beneficial roles of honey polyphenols against some human degenerative diseases: A review. Pharm. Rep..

[12] Al-Waili Salom K., Al-Ghamdi A., Ansari M.J., Al-Waili A., Al-Waili T. (2013). Honey and cardiovascular risk factors, in normal individuals aand in patients with diabetes mellitus or dyslipidemia. J. Med. Food.

[13] Memon M.Q., Kumar A. (2013). The fructose mystery: How bad or good is it?. Pak. J. Pharm. Sci..

[14] O’Neal S.T., Brewster C.C., Bloomquist J.R., Anderson T.D. (2017). Amitraz and its metabolite honey bee cardiac function and tolerance to viral infection. J. Invertebr. Pathol..

[15] Frankel S., Robinson G.E., Berenbaum M.R. (1998). Antioxidnat capacity and correlation characteristics of 14 unifloral honeys. J. Apic. Res..

[16] Gheldof N., Wang X.-H., Engeseth N.J. (2002). Identificaation and quantification of antioxidant components of honeys from various floral sources. J. Agric. Food Chem..

[17] Meda A., Lamien C.E., Romito M., Millogo J., Nacoulma O.G. (2005). Determination of the total phenolic, flavonoid and prolinę contents in Burkina Fasan honey, as well as their radical scavenging activity. Food Chem..

[18] Alvarez-Suarez J.M., Tulipani S., Díaz D., Estevez Y., Romandini S., Giampieri F., Damiani E., Astolfi P., Bompadre S., Battino M. (2010). Antioxidant and antimicrobial capacity of several monofloral Cuban honeys and their correlation with color, polyphenol content and other chemical compounds. Food Chem. Toxicol..

[19] Bogdanov S., Jurendic T., Sieber R., Gallmann P. (2008). Honey for nutrition and health: a review. J. Am. Coll. Nutr..

[20] Saba Z., Suzana M., Anum M.Y. (2013). Honey: Food or medicine. Med. Health.

[21] Rao P.V., Krishnan K.T., Salleh N., Gan S.H. (2016). Kumarathevan Biological and therapeutic effects of honey produced by honey bees and stingless bees: A comparative review. Rev. Bras. Farm..

[22] Solayman M., Islam M.A., Paul S., Ali Y., Khalil M.I., Alam N., Gan S.H. (2016). Physiochemical properties, minerals, trace elements, and heavy metals in honey of different origins: A comprehensive review. Compr. Rev. Food Sci. Food Saf..

[23] Ferreres F., Tomas-Barberan F.A., Soler C., Garcia-Viguera C., Ortiz A., Tomas-Lorente F. (1994). A simple extractive technique for honey flavonoid HPLC analysis. Apidologie.

[24] Yao L., Datta N., Tomas-Barberan F.A., Ferreres F., Martos I., Singanusong R. (2003). Flavonoids, phenolic acids and abscissic acid in Australian and New Zealand Leptospermum honeys. Food Chem..

[25] Arraez-Roman D., Gomez-Caravaca A.M., Gomez-Romero M., Segura-Carretero A., Fernandez-Gutierrez A. (2006). Identification of phenolic compounds in Rosemary honey using solid-phase extraction by capillary electrophoresis-electrospray ionization-mass spectrometry. J. Pharm Biomed. Anal..

[26] Hamdy A.A., Ismail H.M., Al-Ahwal A.M., Gomaa N.F. (2009). Determination of flavonoid and phenolic acid contents of Clover, cotton and citrus floral honeys. J. Egypt. Public Heal. Assoc..

[27] Kennedy D.O., Wightman E.L. (2011). Herbal extracts and phytochemicals: Plant secondary metabolites and the enhancement of human brain function. Adv. Nutr..

[28] Chan C.W., Deadman B.J., Manley-Harris M., Wilkins A.L., Alber D.G., Harry E. (2013). Analysis of the flavonoid component of bioactive New Zealand Manuka (*Leptospermum scoparium*) honey and the isolation, characterization and synthesis of an unusual pyrrole. Food Chem..

[29] Keckes J., Trifkovic J., Andric F., JOvetic M., Tesic Z., Milojkovic-Opsenica D. (2013). Amino acids profile of Serbian unifloral heneys. J. Sci. Food Agric..

[30] Campone L., Piccinelli A.L., Pagano I., Carabetta S., Di Sanzo R., Russo M., Rastrelli L. (2014). Determination of phenolic compounds in honey using dispersive liquid-liquid microextraction. J. Chromatogr. A.

[31] Campillo N., VInas P., Ferez-Melgarejo G., Hernandez-Cordoba M. (2015). Dispersive liquid-liquid microextraction for the determination of flavonoid aglycone compounds in honey using liquid chromatography with diode array detection and time-of-flight mass spectrometry. Talanta.

[32] Petretto G.L., Cossu M., Alamanni M.C. (2015). Phenolic content, antioxidant and physico-chemical properties of Sardinian monofloral honeys. Int. J. Food Sci. Technol..

[33] Akalin H., Bayram M., Anli R.E. (2016). Determination of some individual phenolic compounds and antioxidant capacity of mead produced from different types of honey. J. Inst. Brew..

[34] Kus P.M., Szweda P., Jerkpvic I., Tuberoso C.I. (2016). Activity of Polish unifloral honeys against pathogenic bacteria and its correlation with colour, phenolic content, antioxidant capacity and other paarmeters. Lett. Appl. Microbiol..

[35] Ranneh Y., Ali F., Zarei M., Md Akim A., Hamid H.A., Khazaai H. (2018). Malaysian stingless bee and Tualang honeys: A comparative characterization of total antioxidant capacity and phenolic profile using liquid chromatography-mass spectrometry. LWT-Food Sci. Technol..

[36] Pyrzynska K., Biesaga M. (2009). Analysis of phenolic acids and flavonoids in honey. Trends Anal. Chem..

[37] Fahey J.W., Stephenson K.K. (2002). Pinostrobin from honey and Thai ginger (Boesenbergia pandurata): A potent flavonoid inducer of mammalian phase 2 chemoprotective and antioxidant enzymes. J. Agric. Food Chem..

[38] Al-Mamary M., Al-Meeri A., Al-Habori M. (2002). Antioxidant activities and total phenolics of different types of honey. Nutr. Res..

[39] Kenjeric D., Mandic M.L., Primorac L., Bubalo D., Perl A. (2007). Flavonoid profile of Robinia honeys produced in Croatia. Food Chem..

[40] Spilioti E., Jaakkola M., Tolonen T., Lipponen M., Virtanen V., Chinou I., Kassi E., Karabournioti S., Moutsatsou P. (2014). Phenolic Acid Composition, Antiatherogenic and Anticancer Potential of Honeys Derived from Various Regions in Greece. PLoS ONE.

[41] Ciulu M., Spano N., Pilo M.I., Sanna G. (2016). Recent advances in the analysis of phenolic compounds in unifloral honeys. Molecules.

[42] Ferreres F., Andrade P., Gil M.I., Tomás-Barberán F.A. (1996). Floral nectar phenolics as biochemical markers for the botanical origin of heather honey. Eur. Food Res. Technol..

[43] Schramm D.D., Karim M., Schrader H.R., Holt R.R., Cardetti M., Keen C.L. (2003). Honey with high levels of antioxidants can provide protection to healthy human subjects. J. Agric. Food Chem..

[44] O’Malley T., Langhorne P., Elton R., Stewart C. (1995). Platelet size in stroke patients. Stroke.

[45] Ruggeri Z.M. (2002). Platelets in atherothrombosis. Nat. Med..

[46] Angiolillo D.J., Bernardo E., Sabaté M., Jimenez-Quevedo P., Costa M.A., Palazuelos J., Hernandez-Antolín R., Moreno R., Escaned J., Alfonso F. (2007). Impact of Platelet Reactivity on Cardiovascular Outcomes in Patients with Type 2 Diabetes Mellitus and Coronary Artery Disease. J. Am. Coll. Cardiol..

[47] Daskalova E., Delchev S., Peeva Y., Vladimirova-Kitova L., Kratchanova M., Kratchanov C., Denev P. (2015). Antiatherogenic and Cardioprotective Effects of Black Chokeberry (Aronia melanocarpa) Juice in Aging Rats. Evidence-Based Complement. Altern. Med..

[48] Ahmed A., Alam Khan R., Azim M.K., Saeed S.A., Mesaik M.A., Ahmed S., Imran I. (2011). Effect of natural honey on human platelets and blood coagulation proteins. Pak. J. Pharm. Sci..

[49] Rossiter K., Cooper A.J., Voegeli D., Lwaleed B.A. (2010). Honey promotes angiogenetic activity in the rat aortic ring assay. J. Wound Care.

[50] Kim T.H., Lee K.M., Hong N.D., Jung Y.S. (2016). Anti-platelet and anti-thrombotic effect of a traditional herbal medicine Kyung-Ok-Ko. J. Ethnopharmacol..

[51] James P., Kamara H., Bah A.J., Steel A., Wardle J. (2018). Herbal medicine use among hypertensive patients attending public and private health facilities in Freetown Sierra Leone. Complement. Ther. Clin. Pr..

[52] Rakha M.K., Nabil Z.I., Hussein A.A. (2008). Cardioactive and vasoactive effects of natural wild honey against cardiac malperformance induced by hyperadrenergic activity. J. Med. Food.

[53] Khalil M.I., Tanvir E.M., Afroz R., Sulaiman S.A., Gan S.H. (2015). Cardioprotective effects of Tualang honey: Melioration of cholesterol and cardiac enzymes levels. Biomed. Res. Int..

[54] Afroz R., Tanvir E.M., Karim N., Hossain M.S., Alam N., Gan S.H., Khalil M.I. (2016). Sundarban honey confers protection against isoproterenol-induced myocardial infarction in Wistar rats. Biomed. Res. Int..

[55] Islam A., Khalil I., Islam N., Moniruzaman M., Mottalib A., Sulaiman S.A., Gan S.H. (2012). Physicochemical and antioxidant properties of Bangladeshi honeys stored for more than one year. BMC Complement. Altern. Med..

[56] Erejuwa O.O., Nwobodo N.N., Akpan J.L., Okorie U.A., Ezeonu C.T., Ezeokpo B.C., Nwadike K.I., Erhiano E., Wahab M.S.A., Sulaiman S.A. (2016). Nigerian honey ameliorates hyperglycemia and dyslipidemia in alloxan-induced diabetic rats. Nutrients.

[57] Rasad H., Entezari M.H., Ghadiri E., Mahaki B., Pahlavani N. (2018). The effect of honey consumption compared with sucrose on lipid profile in young healthy subjects (randomized clinical trial). Clin. Nutr. ESPEN.

[58] Raatz S.K., Johnson L.K., Picklo M.J. (2015). Consumption of honey, sucrose, and high-fructose corn syrup produces similar metabolic effects in glucose-tolerant and –intolerant individuals. J. Nutr..

[59] Khalil M.I., Tanvir E.M., Afroz R., Sulaiman S.A., Gan S.H. (2015). Cardioprotective effects of Tualang honey: Amelioration of cholesterol and cardiac enzymes levels. Biomed. Res. Int..

[60] Gutiérrez-Venegas G., Ventura-Arroyo J.A., Arreguín-Cano J.A., Ostoa-Pérez M.F. (2014). Flavonoids inhibit iNOS production via mitogen activated proteins in lipoteichoic acid stimulated cardiomyoblasts. Int. Immunopharmacol..

[61] Maaliki D., Shaito A.A., Pintus G., El-Yazbi A., Eid A.H. (2019). Flavonoids in hypertension: A brief review of the underlying mechanisms. Curr. Opin. Pharm..

[62] Carlos A.U., David H., Carmen G. (2011). Role of honey polyphenols in health. J. Apiproduct. Apimedical. Sci..

[63] Khalil M.I., Alam N., Moniruzzaman M., Sulaiman S.A., Gan S.H. (2011). Phenolic acid composition and antioxidant properties of Malaysian honeys. J. Food Sci..

[64] Tuberoso C.I.G., Boban M., Bifulco E., Budimir D., Pirisi F.M. (2013). Antioxidant capacity and vasodilatory properties od Mediterranean food: The case of Cannonau wine, myrtle berries liqueur and strawberry-tree honey. Food Chem..

[65] Guerrero J., Lozano M., Castillo J., Benavente-Garcia O., Vincente V., Rivera J. (2005). Flavonoids inhibit platelet function through binding to the thromboxane A2 receptor. J. Thromb. Haemost..

[66] Anghel N., Cotoraci C., Ivan A., Suciu M., Herman H., Balta C., Nicolescu L., Olariu T., Galajda Z., Ardelean A. (2015). Chrysin attenuates cardiomyocyte apoptosis and loss of intermediate filaments in a mouse model of mitoxantrone cardiotoxicity. Histol. Histopathol..

[67] Farkhondeh T., Samarghandian S., Bafandeh F. (2019). The Cardiovascular Protective Effects of Chrysin: A Narrative Review on Experimental Researches. Cardiovasc. Hematol. Agents Med. Chem..

[68] Liu G., Xie W., He A., Da X., Liang M., Yao G., Xiang J., Gao C., Ming Z. (2016). Antiplatelet activity of chrysin via inhibiting platelet αIIbβ3-mediated signaling pathway. Mol. Nutr. Food Res..

[69] Ravishankar D., Salamah M., Attina A., Pothi R., Vallance T.M., Javed M., Williams H.F., Alzahrani E.M.S., Kabova E., Vaiyapuri R. (2017). Ruthenium-conjugated chrysin analogues modulate platelet activity, thrombus formation and haemostasis with enhanced efficacy. Sci. Rep..

[70] Dabeek W.M., Marra M.V. (2019). Dietary quercetin and kaempferol: Bioavailability and potential cardiovascular-related bioactivity in humans. Nutrients.

[71] Yamagata K. (2019). Polyphenols regulate endothelial functions and reduce the risk of cardiovascular disease. Curr. Pharm. Des..

[72] Sanchez M., Galisteo M., Vera R., Villar I.C., Zarzuelo A., Tamargo J., Perez-Vizcaino F., Duarte J. (2006). Quercetin downregulates NADPH oxidase, increases eNOS activity and prevents endothelial dysfunction in spontaneously hypertensive rats. J. Hypertens..

[73] Carlstrom J., Symons J.D., Wu T.C., Bruno R.S., Litwin S.E., Jalili T. (2007). A quercetin supplemented diet does not prevent cardiovascular complications in spontaneously hypertiensive rats. J. Nutr..

[74] Duarte J., Galisteo M., Ocete M.A., Perez-Vizcaino F., Zarzeulo A., Tamargo J. (2001). Effects of chronić quercetin treatment on hepatic sttus of spontaneously hypertensive rats. Mol. Cell Biochem..

[75] Hanasaki Y., Ogawa S., Fukui S. (1994). The coreelation between active oxygens scavenging and antioxidative effects of flavonoids. Free Radic. Biol. Med..

[76] Min Z., Yangchun L., Yuguan W., Changying Z. (2019). Quercetin inhibition of myocardial fibrosis through regulating MAPK signaling pathway via ROS. Pak. J Pharm. Sci..

[77] Wu X.J., Zhou X.B., Chen C., Mao W. (2019). Systematic investigation of quercetin for treating cardiovascular disease based on network pharmacology. Comb. Chem. High. Throughput Screen.

[78] Nie J., Zhang L., Zhao G., Du X. (2019). Quercetin reduces atherosclerotic lesions by altering the but microbiota and reducing atherogenic lipid metabolites. J. Appl. Microbiol..

[79] Kong L., Luo C., Li X., Zhou Y., He H. (2013). The anti-inflammatory effect of kaempferol on early atherosclerosis in high cholesterol fed rabbits. Lipids Health Dis..

[80] Li L., Luo W., Qian Y., Zhu W., Qian J., Li J., Jin Y., Xu X., Liang G. (2019). Luteolin protects against diabetic cardiomyopathy by inhibition NF-κB-mediated inflammation and activating the Nrf2-mediated antioxidant responses. Phytomed.

[81] Oyagbemi A.A., Omobowale T.O., Ola-Davies O.E., Aeenuga E.R., Ajibade T.O., Adejumobi O.A., Afolabi J.M., Ogunpolu B.S., Falayi O.O., Saba A.B. (2018). Luteolin-mediated Kim-1/NF-κB/Nrf2 signaling pathways protects sodium fluoride-induced hypertension and cardiovascular complications. Biofactors.

[82] Ou H.C., Pandey S., Hung M.Y., Huang S.H., Hsu P.T., Day C.H., Pai P., Viswanadha V.P., Kuo W.W., Huang C.Y. (2019). Luteolin: A natural flavonoid enhances the survival of HUVECs against oxidative stress by modulating AMPK/PKC pathway. Am. J. Chin. Med..

[83] Jiang D., Li D., Wu W. (2013). Inhibitory Effects and Mechanisms of Luteolin on Proliferation and Migration of Vascular Smooth Muscle Cells. Nutrients.

[84] Lungkaphin A., Pongchaidecha A., Palee S., Arjinajarn P., Pompomon W., Chattipakorn N. (2015). Pinocembrin reduces cardiac arrhythmia and infarct size in rats subjected to acute myocardial ischemia/reperfusion. Appl. Physiol. Nutr. Metab..

[85] Sang H., Yuan N., Yao S., Li F., Wang J., Fang Y., Qin S. (2012). Inhibitory effect of the combination therapy of simvastatin and pinocembrin on atherosclerosis in ApoE-deficient mice. Lipids Health Dis..

[86] Wang H.B., Huang S.H., Xu M., Yang J., Yang J., Liu M.X., Wan C.X., Liao H.H., Fan D., Tang Q.Z. (2019). Galangin ameliorates cardiac remodeling via the MEK1/2-ERK1/2 and PI3K-AKT pathways. J. Cell Physiol..

[87] Lafay S., Gueux E., Rayssinguier Y., Mazur A., Remesy C., Scalbert A. (2005). Caffeic acid inhibits oxidative stress and reduces hypercholesterolemia induced by iron overload in rats. Int. J. Vitam. Nutr. Res..

[88] Al-Waili N., SAlom K., Al-Ghamdi A., Ansari M.J. (2012). Antibiotic, pesticide, and microbial contaminants of honey: Human health hazards. Sci. World J..

[89] Yaylaci S., Kocayigit I., Aydin E., Osken A., Genc A.B., Cakar M.A., Tamer A. (2014). Clinical and laboratory findings in mad honey poisoning: A single center experience. Niger. J. Clin. Pr..

[90] Dur A., Sonmez E., Civelek C., Tutkdogan K.A., Vatankulu M.A., Sogut O. (2014). Mad honey intoxication mimicking acute coronary syndrome. J. Pak. Med. Assoc..

[91] Karabag T., Sayin R., Yavuz N., Aktop Z. (2015). Type 2 myocardial infarction after ingestion of md honey in a patient with normal coronary arteries. Korean J. Intern. Med..

[92] Erenler A.K. (2016). Cardiac effects of mad honey poisoning and its management in emergency department: A review from Turkey. Cardiovasc. Toxicol..

[93] Aazza S., Lyoussi B., Antenues D., Miguel M.G. (2013). Physico-chemical characterization and antioxidant activity of commercial Portuguese honeys. J. Food Sci..

[94] Aazza S., Lyoussi B., Antenues D., Miguel M.G. (2014). Physico-chemical characterization and antioxidant activity of 17 commercial Moroccan honeys. Int. J. Food Nutr..

[95] Karabagias I.K., Dimitriou E., Kontakos S., Kontominas M.G. (2016). Phenolic profile, colour intensity, and radical scavenging activity of Greek unifloral honeys. Eur. Food Res. Technol..

[96] Abdeltaif S.A., SirElkhatim K.A., Hassan A.B. (2018). Estimation of phenolic and flavonoid compounds and antioxidant activity of spent coffee and black tea (processing) waste for potential recovery and reuse in Sudan. Recycling.

